# Supplementary material for: Effects of ethnicity and geography on the fecal microbiota and dietary habits of Tibeto-Burman hill tribes in Northern Thailand
Source: PLoS One. 2025 Oct 6;20(10):e0332108. doi: 10.1371/journal.pone.0332108 (PMC12500092; doi:10.1371/journal.pone.0332108)
Supplement: S1 Text — S1 Fig. Partial least squares discriminant analysis (PLS-DA) of gut microbiota across ethnic groups. Discriminant analysis identifying key features (microbial taxa) associated with each ethnic group in Chiang Mai province, where the median values are maximized along Component 1 (A) and Component 2 (B). (C) Discriminant analysis identifying key features (microbial taxa) associated with each ethnic group in Chiang Rai province, where the median values are maximized along Component 1. Bar lengths represent the loading weights, reflecting the contribution of each taxon (ranked from bottom to top). Red = AkhaCM; Light red = AkhaCR; Blue = LahuCM; Light blue = LahuCR; Light purple = LisuCR; CM = Chiang Mai; CR = Chiang Rai. S2 Fig. Multiple factor analysis (MFA) integrating gut microbiota and dietary habits between ethnic groups in Chiang Mai Province. (A) Bar plot showing the contribution of groups to Dimensions 1 and 2 (Dim 1 and Dim 2). (B) Correlation plot illustrating the relationship between dietary habits and dimensions. (C) Correlation circle plot showing the association between quantitative variables (microbial taxa) and dimensions. (D) Factor map displaying individual profiles grouped by ethnicity. Red = AkhaCM; Blue = LahuCM; CM = Chiang Mai; Dim = dimension. S3 Fig. Multiple factor analysis (MFA) integrating gut microbiota and dietary habits across ethnic groups in Chiang Rai Province. (A) Bar plot showing the contribution of groups to Dimensions 1 and 2 (Dim 1 and Dim 2). (B) Correlation plot illustrating the relationship between dietary habits and dimensions. (C) Correlation circle plot showing the association between quantitative variables (microbial taxa) and dimensions. (D) Factor map displaying individual profiles grouped by ethnicity. Light red = AkhaCR; Light blue = LahuCR; Light purple = LisuCR; CR = Chiang Rai; Dim = dimension. S4 Fig. Multiple factor analysis (MFA) integrating gut microbiota and dietary habits, and participant characteristics betwee [file pone.0332108.s001.zip › S6_Fig.pdf]

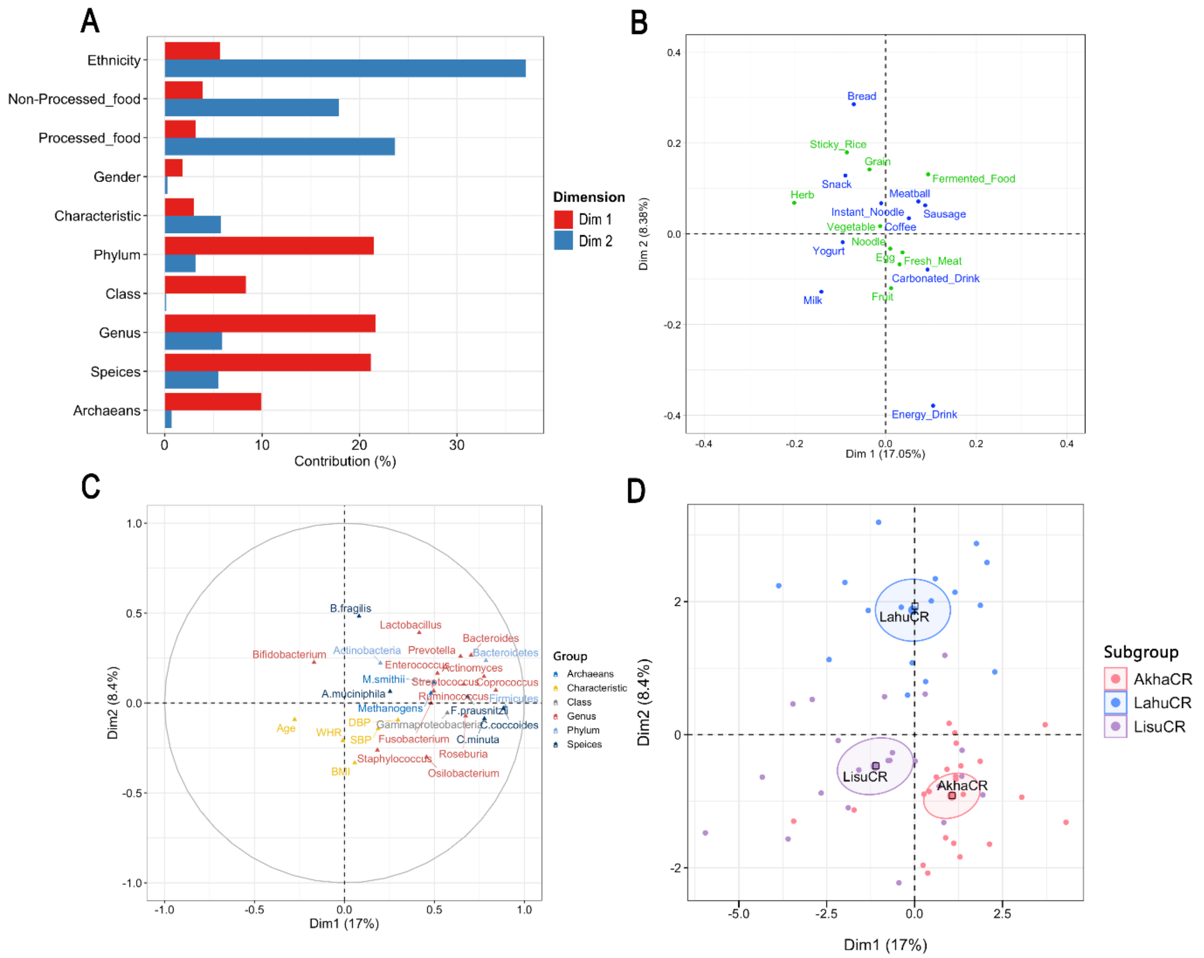

S6 Fig. Multiple factor analysis (MFA) integrating gut microbiota and dietary habits, and participant characteristics between ethnic groups in Chiang Rai Province. (A) Bar plot showing the contribution of groups to Dimensions 1 and 2 (Dim 1 and Dim 2). (B) Correlation plot illustrating the relationship between dietary habits and dimensions. (C) Correlation circle plot showing the association between quantitative variables (microbial taxa and participant characteristics) and dimensions. (D) Factor map displaying individual profiles grouped by ethnicity. Light red = AkhaCR; Light blue = LahuCR; Light purple = LisuCR; CR = Chiang Rai; Dim = dimension.
