# Supplementary material for: Effects of ethnicity and geography on the fecal microbiota and dietary habits of Tibeto-Burman hill tribes in Northern Thailand
Source: PLoS One. 2025 Oct 6;20(10):e0332108. doi: 10.1371/journal.pone.0332108 (PMC12500092; doi:10.1371/journal.pone.0332108)
Supplement: S2 Text — S1 Table. Primer pairs targeting bacterial 16S rRNA genes. S2 Table. Mean differences in gut microbiota abundance between ethnic groups within Chiang Mai Province. S3 Table. Mean differences in gut microbiota abundance between ethnic groups within Chiang Rai Province. S4 Table. Median differences in dietary consumption frequencies between ethnic groups within Chiang Mai Province. S5 Table. Median differences in dietary consumption frequencies between ethnic groups within Chiang Rai Province. S6 Table. Significant Spearman’s rank Correlations (p < 0.05) between gut microbiota and dietary habits in Chiang Mai province. S7 Table. Significant Spearman’s rank Correlations (p < 0.05) between gut microbiota and dietary habits in Chiang Rai province. S8 Table. Mean differences in gut microbiota abundance between geographic locations within the Akha ethnic group. S9 Table. Mean differences in gut microbiota abundance between geographic locations within the Lahu ethnic group. S10 Table. Median differences in dietary consumption frequencies between geographic locations within the Akha ethnic group. S11 Table. Median differences in dietary consumption frequencies between geographic locations within the Lahu ethnic group. (ZIP) [file pone.0332108.s002.zip › S1_Table.pdf]

S1 Table. Primer pairs targeting bacterial 16S rRNA genes.

Target[a]	Primer sequence (5′—>3′)[b]	Product size (bp)	Ta	Reference
Actinobacteria *(P)*	F: TACGGCCGCAAGGCTA<br>R: TCRTCCCCACCTTCCTCCG	170	64	De Gregoris et al., 2011
Bacteroidetes *(P)*	F: CRAACAGGATTAGATACCCT<br>R: GGTAAGGTTCCTCGCGTAT	240	64	De Gregoris et al., 2011
Firmicutes *(P)*	F: TGAAACTYAAAGGAATTGACG<br>R: ACCATGCACCACCTGTC	200	64	De Gregoris et al., 2011
Gammaproteobacteria (C)	F: TCGTCAGCTCGTGTYGTGA<br>R: CGTAAGGGCCATGATG	170	53	De Gregoris et al., 2011
Actinomyces spp. (G)	F: CGGGTTGTRAACCTYTTTC<br>R: GTAACGGCCCAGWRACCC	325	57	Chumponsuk, 2017
Bacteroides spp. (G)	F: GAAGGTCCCCCACATTG<br>R: CGCKACTTGGCTGGTTCAG	103	64	Bartosch et al., 2004; Ramirez-Farias et al., 2009
Bifidobacterium spp. (G)	F: TCGCGTCYGGTGTGAAAG<br>R: GGTGTTCTTCCCGATATCTACA	601	64	Matsuki et al., 2002; Rinttilä et al., 2004
Coprococcus spp. (G)	F: CAACCCCGGRACKGCTTT<br>R: CCATGCACCACCTGTCWCYR	440	65	Noontan, 2017
Enterococcus spp. (G)	F: CCCTTATTGTTAGTTGCCATCATT<br>R: ACTCGTTGTACTTCCCATTGT	144	56	Rinttilä et al., 2004
Fusobacterium spp. (G)	F: CGGGTGAGTAACGCGTAAAG<br>R: GCCGTGTCTCAGTCCCCT	208	55	Walter et al., 2002
Lactobacillus group (G)	F: AGCAGTAGGGAATCTTCCA<br>R: CACCGCTACACATGGAG	341	56	Walter et al., 2001; Heilig et al., 2002
Osilobacterium spp. (G)	F: ACGGTACCCCTTGAATAAGCC<br>R: TCCCCGCACACCTAGTATTG	359	64	Mackie et al., 2003; Yanagita et al., 2003
Prevotella spp. (G)	F: GCCGCGGTAATACGGAAGG<br>R: CTAATCCTGTTYGATACCCGCAC	267	56	Jinatham, 2015
Roseburia spp.	F: GCGGTRCGGCAAGTCTGA<br>R: CCTCCGACACTCTAGTMCGAC	80	55	Walker et al., 2005; Ramirez-Farias et al., 2009
Ruminococcus spp. (G)	F: GGCGGCYTRCTGGGCTTT<br>R: CCAGGTGGATWACTTATTGTGTTAA	156	63	Ramirez-Farias et al., 2009

Note: [a]P=phylum, C=class and G=genus, S= Species, A= Archaeans; [b]F and R represent forward (F) and reverse (R) primers, respectively.

S1 Table. Primer pairs targeting bacterial 16S rRNA genes (continued).

Target[a]	Primer sequence (5′—>3′)[b]	Product size (bp)	Ta	Reference
Streptococcus spp. (G)	F: CGATACATAGCCGACCTGAGA<br>R: CACTCTCCCCTYYTGCAC	395	69	Chumponsuk, 2017
Staphylococcus spp. (G)	F: CGTCTTGACGGTACCTAATC<br>R: CTCCATATCTCTGCGCATTTC	237	60	Kullawong, 2015
A.muciniphila (S)	F: CAGCACGTGAAGGTGGGGAC<br>R: CCTTGCGGTTGGCTTCAGAT	349	60	Collado et al., 2007
B.fragilis (S)	F: TCRGGAAGAAAGCTTGCT<br>R: CATCCTTTACCGGAATCCT	162	60	Tong et al., 2011
C.minuta (S)	F: GTAATACGTAGGGAGCAAGC<br>R: CCCTCTCCTGTACTCAAGTC	145	55	Jinatham, 2015
Cl. coccoides (S)	F: CGGTACCTGACTAAGAAGC<br>R: AGTTTYATTCTTGCGAACG	429	60	Rinttilä et al., 2004
F.prausnitzii (S)	F: GGAGGAAGAAGGTCTTCGG<br>R: AATTCCGCCTACCTCTGCACT	247	63	Wang et al., 1996; Ramirez-Farias et al., 2009
Methanogens (A)	F: GGATTAGATACCCSGGTAGT<br>R: GTTGARTCCAATTAAACCGCA	190	56	Hook et al., 2009
M.smithii (A)	F: CTCCCAGGGTAGAGGTGAAA<br>R: CCGGGTATCTAATCCGGTTC	123	63	Raskin et al., 1994

Note: [a]P=phylum, C=class and G=genus, S= Species, A= Archaeans; [b]F and R represent forward and reverse primers, respectively.

References

Bacchetti De Gregoris T, Aldred N, Clare AS, et al. (2011) Improvement of phylum- and class-specific primers for real-time PCR quantification of bacterial taxa. J Microbiol Methods 86:351–356. https://doi.org/https://doi.org/10.1016/j.mimet.2011.06.010

Bartosch S, Fite A, Macfarlane GT, et al. (2004) Characterization of Bacterial Communities in Feces from Healthy Elderly Volunteers and Hospitalized Elderly Patients by Using Real-Time PCR and Effects of Antibiotic Treatment on the Fecal Microbiota. Appl Environ Microbiol 70:3575–3581. https://doi.org/10.1128/AEM.70.6.3575-3581.2004

Collado MC, Derrien M, Isolauri E, et al. (2007) Intestinal Integrity and Akkermansia muciniphila , a Mucin-Degrading Member of the Intestinal Microbiota Present in Infants, Adults, and the Elderly. Appl Environ Microbiol 73. https://doi.org/10.1128/AEM.01477-07

Heilig HGHJ, Zoetendal EG, Vaughan EE, et al. (2002) Molecular Diversity of Lactobacillus spp. and Other Lactic Acid Bacteria in the Human Intestine as Determined by Specific Amplification of 16S Ribosomal DNA. Appl Environ Microbiol 68:114–123. https://doi.org/10.1128/AEM.68.1.114-123.2002

Matsuki T, Watanabe K, Fujimoto J, et al. (2002) Development of 16S rRNA-Gene-Targeted Group-Specific Primers for the Detection and Identification of Predominant Bacteria in Human Feces. Appl Environ Microbiol 68:5445–5451. https://doi.org/10.1128/AEM.68.11.5445-5451.2002

Ramirez-Farias C, Slezak K, Fuller Z, et al. (2008) Effect of inulin on the human gut microbiota: stimulation of Bifidobacterium adolescentis and Faecalibacterium prausnitzii. Br J Nutr 101:541–550. https://doi.org/10.1017/S0007114508019880

Raskin, L., Stromley, J. M., Rittmann, B. E., & Stahl, D. A. (1994). Group-specific 16S rRNA hybridization probes to describe natural communities of methanogens. Applied and environmental microbiology, 60(4), 1232-1240.

Rinttila T, Kassinen A, Malinen E, et al. (2004) Development of an extensive set of 16S rDNA-targeted primers for quantification of pathogenic and indigenous bacteria in faecal samples by real-time PCR. J Appl Microbiol 97:1166–1177. https://doi.org/10.1111/j.1365-2672.2004.02409.x

Stevenson DM, Weimer PJ (2007) Dominance of Prevotella and low abundance of classical ruminal bacterial species in the bovine rumen revealed by relative quantification real-time PCR. Appl Microbiol Biotechnol 75:165–174. https://doi.org/10.1007/s00253-006-0802-y

Walker AW, Duncan SH, McWilliam Leitch EC, et al. (2005) pH and Peptide Supply Can Radically Alter Bacterial Populations and Short-Chain Fatty Acid Ratios within Microbial Communities from the Human Colon. Appl Environ Microbiol 71. https://doi.org/10.1128/AEM.71.7.3692-3700.2005

Walter J, Hertel C, Tannock GW, et al. (2001) Detection of Lactobacillus, Pediococcus, Leuconostoc, and Weissella Species in Human Feces by Using Group-Specific PCR Primers and Denaturing Gradient Gel Electrophoresis. Appl Environ Microbiol 67:2578–2585. https://doi.org/10.1128/AEM.67.6.2578-2585.2001

Wang RF, Cao WW, Cerniglia CE (1996) PCR detection and quantitation of predominant anaerobic bacteria in human and animal fecal samples. Appl Environ Microbiol 62. https://doi.org/10.1128/aem.62.4.1242-1247.1996
