# Supplementary material for: Effects of ethnicity and geography on the fecal microbiota and dietary habits of Tibeto-Burman hill tribes in Northern Thailand
Source: PLoS One. 2025 Oct 6;20(10):e0332108. doi: 10.1371/journal.pone.0332108 (PMC12500092; doi:10.1371/journal.pone.0332108)
Supplement: S2 Text — S1 Table. Primer pairs targeting bacterial 16S rRNA genes. S2 Table. Mean differences in gut microbiota abundance between ethnic groups within Chiang Mai Province. S3 Table. Mean differences in gut microbiota abundance between ethnic groups within Chiang Rai Province. S4 Table. Median differences in dietary consumption frequencies between ethnic groups within Chiang Mai Province. S5 Table. Median differences in dietary consumption frequencies between ethnic groups within Chiang Rai Province. S6 Table. Significant Spearman’s rank Correlations (p < 0.05) between gut microbiota and dietary habits in Chiang Mai province. S7 Table. Significant Spearman’s rank Correlations (p < 0.05) between gut microbiota and dietary habits in Chiang Rai province. S8 Table. Mean differences in gut microbiota abundance between geographic locations within the Akha ethnic group. S9 Table. Mean differences in gut microbiota abundance between geographic locations within the Lahu ethnic group. S10 Table. Median differences in dietary consumption frequencies between geographic locations within the Akha ethnic group. S11 Table. Median differences in dietary consumption frequencies between geographic locations within the Lahu ethnic group. (ZIP) [file pone.0332108.s002.zip › S3_Table.pdf]

S3 Table. Mean differences in gut microbiota abundance between ethnic groups within Chiang Rai Province.

Bacterial	AkhaCR (n=23)	LahuCR (n=16)	LisuCR (n=22)	*p*-value	Sig.Post hoc (*q*-value)
Firmicutes[c]	9.61±0.38	9.42±0.36	9.26±0.54	0.038*	AkhaCR > LisuCR (0.038*)
Bacteroidetes[c]	10.46±0.52	10.53±0.52	10.05±0.69	0.053	
Actinobacteria[a]	8.42±0.39	8.69±0.55	8.45±0.60	0.241	
Gammaproteobacteria[a]	7.55±0.40	7.33±0.37	7.25±0.41	0.037*	AkhaCR > LisuCR (0.033*)
Bacteroides[c]	11.14±0.63	11.31±0.56	10.93±0.68	0.262	
Prevotella[a]	11.02±0.89	11.51±0.71	10.99±0.87	0.125	
Streptococcus[b]	10.71±0.37	10.78±0.78	10.25±0.71	0.029*	
Enterococcus[a]	8.12±0.48	8.31±0.51	7.92±0.51	0.060	
Roseburia[a]	9.49±0.45	9.22±0.6	9.29±0.62	0.281	
Ruminococcus[c]	9.77±0.37	9.7±0.42	9.32±0.63	0.011*	AkhaCR > LisuCR (0.01*)
Coprococcus[a]	10.72±0.59	10.55±0.75	10.29±0.85	0.156	
Staphylococcus[a]	6.97±0.48	6.78±0.4	7.05±0.37	0.158	
Osilobacterium[c]	8.00±1.24	7.13±2.06	7.36±1.52	0.165	
Fusobacterium[a]	7.22±1.16	6.64±0.88	6.94±0.97	0.229	
Actinomyces[a]	8.64±0.41	8.62±0.51	8.08±0.50	$p < 0.001$***	AkhaCR > LisuCR ($q <$ 0.001***)<br>LahuCR > LisuCR (0.002**)
Bifidobacterium[a]	7.71±0.95	8.32±0.86	8.11±1.08	0.1490	
Lactobacillus[a]	7.97±0.44	8.41±0.63	7.6±0.62	$p < 0.001$***	LahuCR > LisuCR ($q <$ 0.001***)
B.fragilis[c]	7.93±1.14	8.61±1.15	8.1±1.35	0.2280	
C.coccoides[a]	9.47±0.43	9.18±0.46	9.11±0.66	0.0630	
C.minuta[a]	9.69±0.39	9.52±0.52	9.24±0.55	0.0110*	AkhaCR > LisuCR (0.008**)
F.prausnitzii[c]	9.3±0.99	8.99±0.56	8.89±0.5	0.1640	
A.muciniphila[c]	6.39±0.86	6.03±0.5	6.27±1.48	0.5690	
Methanogens[c]	9.87±1.37	9.58±0.36	9.3±0.57	0.0410*	
M.smithii[c]	8.9±0.74	8.9±0.74	9.05±0.78	0.7280	

The relative abundance of gut microbiota is expressed as mean ± standard deviation (SD).
[a] ANOVA with Tukey’s post-hoc test with BH *p*-value correction
[b] Welch’s ANOVA with Games-Howell test with BH *p*-value correction
[c] Kruskal–Wallis test with Dunn’s test with BH *p*-value correction
* $p < 0.05$; **; $p < 0.01$; ***$p < 0.001$
*$q < 0.05$; **$q < 0.01$; ***$q < 0.001$
