# Supplementary material for: Effects of ethnicity and geography on the fecal microbiota and dietary habits of Tibeto-Burman hill tribes in Northern Thailand
Source: PLoS One. 2025 Oct 6;20(10):e0332108. doi: 10.1371/journal.pone.0332108 (PMC12500092; doi:10.1371/journal.pone.0332108)
Supplement: S2 Text — S1 Table. Primer pairs targeting bacterial 16S rRNA genes. S2 Table. Mean differences in gut microbiota abundance between ethnic groups within Chiang Mai Province. S3 Table. Mean differences in gut microbiota abundance between ethnic groups within Chiang Rai Province. S4 Table. Median differences in dietary consumption frequencies between ethnic groups within Chiang Mai Province. S5 Table. Median differences in dietary consumption frequencies between ethnic groups within Chiang Rai Province. S6 Table. Significant Spearman’s rank Correlations (p < 0.05) between gut microbiota and dietary habits in Chiang Mai province. S7 Table. Significant Spearman’s rank Correlations (p < 0.05) between gut microbiota and dietary habits in Chiang Rai province. S8 Table. Mean differences in gut microbiota abundance between geographic locations within the Akha ethnic group. S9 Table. Mean differences in gut microbiota abundance between geographic locations within the Lahu ethnic group. S10 Table. Median differences in dietary consumption frequencies between geographic locations within the Akha ethnic group. S11 Table. Median differences in dietary consumption frequencies between geographic locations within the Lahu ethnic group. (ZIP) [file pone.0332108.s002.zip › S5_Table.pdf]

S5 Table. Median differences in dietary consumption frequencies between ethnic groups within Chiang Rai Province.

Food Item	Median of consumption frequency (times per week)				
	**AkhaCR (n=23)**	**LahuCR (n=16)**	**LisuCR (n=23)**	***p*-value[a]**	**Sig.Post hoc[b] (*q*-value)**
**Non-Processed Foods**	3	2	3	0.024*	AkhaCR > LahuCR (0.020*)
Fresh Meat	6	4.5	6	$p < 0.001$***	AkhaCR > LahuCR (0.001**), LahuCR > LisuCR ($q < 0.001$***)
Egg	4	3	4	0.010*	AkhaCR > LahuCR (0.009**), LahuCR < LisuCR (0.032*)
Grain	2	2	2	0.026*	AkhaCR < LisuCR (0.021*)
Sticky Rice	1	2	2	$p < 0.001$***	AkhaCR < LahuCR ($q < 0.001$***), AkhaCR < LisuCR ($q < 0.001$***)
Rice	6	6	6	1.000	
Noodle	2	2	2	0.405	
Vegetable	6	6	6	0.532	
Fruit	4	4	4	0.238	
Herb	1	1	1	0.172	
Fermented Food	2	2	2	0.870	
**Processed Foods**	2	2	2	0.385	
Milk	1	2	2.5	0.144	
Carbonated Drink	2	2	2	0.736	
Snack	2	2	2	0.253	
Bread	1	2	2	0.020*	AkhaCR < LahuCR (0.017*)
Noodle	2	2	2	0.549	
Energy Drink	2	1	1.5	0.214	
Coffee	1	2	1	0.391	
Yogurt	1	1	2	0.214	
Meatball	2	2	2	0.185	
Sausage	2	2	2	0.512	

[a]Kruskal–Wallis test
[b] Dunn’s test Benjamini–Hochberg method
*$p < 0.05$; **$p < 0.01$; ***$p < 0.001$
*$q < 0.05$; **$q < 0.01$; ***$q < 0.001$
