# Supplementary material for: Effects of ethnicity and geography on the fecal microbiota and dietary habits of Tibeto-Burman hill tribes in Northern Thailand
Source: PLoS One. 2025 Oct 6;20(10):e0332108. doi: 10.1371/journal.pone.0332108 (PMC12500092; doi:10.1371/journal.pone.0332108)
Supplement: S2 Text — S1 Table. Primer pairs targeting bacterial 16S rRNA genes. S2 Table. Mean differences in gut microbiota abundance between ethnic groups within Chiang Mai Province. S3 Table. Mean differences in gut microbiota abundance between ethnic groups within Chiang Rai Province. S4 Table. Median differences in dietary consumption frequencies between ethnic groups within Chiang Mai Province. S5 Table. Median differences in dietary consumption frequencies between ethnic groups within Chiang Rai Province. S6 Table. Significant Spearman’s rank Correlations (p < 0.05) between gut microbiota and dietary habits in Chiang Mai province. S7 Table. Significant Spearman’s rank Correlations (p < 0.05) between gut microbiota and dietary habits in Chiang Rai province. S8 Table. Mean differences in gut microbiota abundance between geographic locations within the Akha ethnic group. S9 Table. Mean differences in gut microbiota abundance between geographic locations within the Lahu ethnic group. S10 Table. Median differences in dietary consumption frequencies between geographic locations within the Akha ethnic group. S11 Table. Median differences in dietary consumption frequencies between geographic locations within the Lahu ethnic group. (ZIP) [file pone.0332108.s002.zip › S6_Table.pdf]

S6 Table. Significant Spearman's rank Correlations (p < 0.05) between gut microbiota and dietary habits in Chiang Mai province.

Taxa		Associated diets	Dietary group	Correlation levels	Study group (s)
Phylum	Firmicutes	Carbonated drink	Processed food	Moderate[a]	AkhaCM
	Bacteroidetes	Egg	Non-processed food	Moderate[b]	AkhaCM
		Noodle	Non-processed food	Moderate[b]	LahuCM
		Carbonated drink	Processed food	Moderate[a]	AkhaCM
		Energy drink	Processed food	Moderate[a]	AkhaCM
	Actinobacteria	Carbonated drink	Processed food	Moderate[b]	LahuCM
		Bread	Processed food	Moderate[a]	LahuCM
		Instant noodle	Processed food	Moderate[a]	AkhaCM
Genus	*Bacteroides*	Yogurt	Processed food	Moderate[b]	AkhaCM
	Prevotella	Sticky rice	Non-processed food	Moderate[a]	AkhaCM
	Streptococcus	Egg	Non-processed food	Moderate[b]	AkhaCM
		Egg	Non-processed food	Moderate[b]	LahuCM
		Carbonated drink	Processed food	Moderate[a]	AkhaCM
	Enterococcus	Energy drink	Processed food	Moderate[a]	AkhaCM
	Roseburia	Fresh Meat	Non-processed food	Moderate[b]	LahuCM
		Fermented food	Non-processed food	Moderate[a]	AkhaCM
		Energy drink	Processed food	Moderate[a]	AkhaCM
	Ruminococcus	Fresh Meat	Non-processed food	Moderate[b]	LahuCM
		Grain	Non-processed food	Moderate[a]	LahuCM
	Coprococcus	Fresh Meat	Non-processed food	Moderate[b]	LahuCM
	Staphylococcus	Herb	Non-processed food	Moderate[b]	AkhaCM
		Milk	Processed food	Moderate[b]	AkhaCM
	Osilobacterium	Fresh Meat	Non-processed food	Moderate[b]	LahuCM
		Egg	Non-processed food	Moderate[b]	AkhaCM
		Vegetable	Non-processed food	Moderate[b]	AkhaCM
	Fusobacterium	Noodle	Non-processed food	Moderate[a]	AkhaCM
		Vegetable	Non-processed food	Moderate[b]	AkhaCM
		Sausage	Processed food	Moderate[b]	LahuCM
	Actinomyces	Fresh Meat	Non-processed food	Moderate[b]	LahuCM
		Milk	Processed food	Moderate[a]	AkhaCM
	Bifidobacterium	Egg	Non-processed food	Moderate[a]	LahuCM
		Herb	Non-processed food	Moderate[b]	LahuCM
		Instant noodle	Processed food	Moderate[a]	AkhaCM
		Milk	Processed food	Moderate[a]	AkhaCM
	Lactobacillus	Egg	Non-processed food	Moderate[b]	LahuCM
		Fermented food	Non-processed food	Moderate[a]	AkhaCM

[a]Positive correlation; [b]Negative correlation

S6 Table. Significant Spearman's rank Correlations ($p < 0.05$) between gut microbiota and dietary habits in Chiang Mai province (continued).

Taxa		Associated diets	Dietary group	Correlation levels	Study group (s)
Species	*B.fragilis*	Egg	Non-processed food	Moderate[b]	AkhaCM
	C.coccoides	Carbonated drink	Processed food	Moderate[a]	AkhaCM
	C.minuta	Fresh Meat	Non-processed food	Strong[b]	LahuCM
		Fermented food	Non-processed food	Moderate[a]	AkhaCM
	F.prausnitzii	Herb	Non-processed food	Moderate[a]	AkhaCM
		Carbonated drink	Processed food	Moderate[a]	AkhaCM
	A.muciniphila	Egg	Non-processed food	Moderate[b]	AkhaCM
		Coffee	Processed food	Moderate[a]	AkhaCM
	Methanogens	Fresh Meat	Non-processed food	Moderate[b]	LahuCM
	M.smithii	Snack	Processed food	Moderate[b]	AkhaCM

[a]Positive correlation; [b]Negative correlation
