# Supplementary material for: Effects of ethnicity and geography on the fecal microbiota and dietary habits of Tibeto-Burman hill tribes in Northern Thailand
Source: PLoS One. 2025 Oct 6;20(10):e0332108. doi: 10.1371/journal.pone.0332108 (PMC12500092; doi:10.1371/journal.pone.0332108)
Supplement: S2 Text — S1 Table. Primer pairs targeting bacterial 16S rRNA genes. S2 Table. Mean differences in gut microbiota abundance between ethnic groups within Chiang Mai Province. S3 Table. Mean differences in gut microbiota abundance between ethnic groups within Chiang Rai Province. S4 Table. Median differences in dietary consumption frequencies between ethnic groups within Chiang Mai Province. S5 Table. Median differences in dietary consumption frequencies between ethnic groups within Chiang Rai Province. S6 Table. Significant Spearman’s rank Correlations (p < 0.05) between gut microbiota and dietary habits in Chiang Mai province. S7 Table. Significant Spearman’s rank Correlations (p < 0.05) between gut microbiota and dietary habits in Chiang Rai province. S8 Table. Mean differences in gut microbiota abundance between geographic locations within the Akha ethnic group. S9 Table. Mean differences in gut microbiota abundance between geographic locations within the Lahu ethnic group. S10 Table. Median differences in dietary consumption frequencies between geographic locations within the Akha ethnic group. S11 Table. Median differences in dietary consumption frequencies between geographic locations within the Lahu ethnic group. (ZIP) [file pone.0332108.s002.zip › S7_Table.pdf]

S7 Table. Significant Spearman's rank Correlations (p < 0.05) between gut microbiota and dietary habits in Chiang Rai province.

Taxa		Associated diets	Dietary group	Correlation levels	Study group (s)
Phylum	Firmicutes	Fresh Meat	Non-processed food	Moderate[a]	AkhaCR
		Vegetable	Non-processed food	Moderate[b]	LahuCR
		Yogurt	Processed food	Moderate[a]	LahuCR
	Bacteroidetes	Fresh Meat	Non-processed food	Moderate[a]	AkhaCR
		Vegetable	Non-processed food	Moderate[b]	LahuCR
		Carbonated drink	Processed food	Moderate[a]	LisuCR
	Actinobacteria	Herb	Non-processed food	Moderate[a]	AkhaCR
		Instant noodle	Processed food	Moderate[a]	LahuCR
		Sausage	Processed food	Moderate[a]	LahuCR
Class	*Gammaproteobacteria*	Fresh Meat	Non-processed food	Moderate[a]	AkhaCR
		Egg	Non-processed food	Moderate[b]	AkhaCR
		Grain	Non-processed food	Moderate[b]	LahuCR
		Milk	Processed food	Strong[a]	LahuCR
Genus	*Bacteroides*	Fresh Meat	Non-processed food	Moderate[a]	AkhaCR
		Grain	Non-processed food	Moderate[a]	LisuCR
		Noodle	Non-processed food	Moderate[a]	LisuCR
		Herb	Non-processed food	Moderate[b]	LisuCR
		Carbonated drink	Processed food	Moderate[a]	LisuCR
	Prevotella	Fresh Meat	Non-processed food	Moderate[a]	AkhaCR
		Yogurt	Processed food	Moderate[a]	LahuCR
	Streptococcus	Coffee	Processed food	Moderate[a]	LisuCR
	Enterococcus	Sticky rice	Non-processed food	Moderate[b]	LisuCR
		Instant noodle	Processed food	Strong[b]	LisuCR
	Roseburia	Fresh Meat	Non-processed food	Moderate[a]	AkhaCR
		Bread	Processed food	Moderate[a]	AkhaCR
	Ruminococcus	Bread	Processed food	Moderate[b]	AkhaCR
	Staphylococcus	Fresh Meat	Non-processed food	Moderate[a]	AkhaCR
		Sticky rice	Non-processed food	Moderate[b]	LisuCR
		Milk	Non-processed food	Moderate[b]	LisuCR
		Yogurt	Processed food	Strong[a]	LahuCR
	Osilobacterium	Egg	Non-processed food	Moderate[b]	LisuCR
		Meatball	Processed food	Moderate[b]	AkhaCR
		Sausage	Processed food	Moderate[b]	AkhaCR
	Fusobacterium	Fruit	Non-processed food	Strong[b]	LahuCR
		Milk	Non-processed food	Moderate[b]	LisuCR

[a] Positive correlation; [b]Negative correlation

S7 Table. Significant Spearman's rank Correlations ($p < 0.05$) between gut microbiota and dietary habits in Chiang Rai province (continued).

Taxa		Associated diets	Dietary group	Correlation levels	Study group (s)
Genus	*Actinomyces*	Fresh Meat	Non-processed food	Moderate[a]	AkhaCR
		Vegetable	Non-processed food	Moderate[b]	LahuCR
		Carbonated drink	Processed food	Moderate[a]	LisuCR
		Snack	Processed food	Moderate[b]	AkhaCR
		Bread	Processed food	Moderate[a]	LisuCR
	Lactobacillus	Milk	Processed food	Moderate[a]	LahuCR
Species	*B.fragilis*	Grain	Non-processed food	Moderate[a]	LisuCR
		Noodle	Non-processed food	Moderate[b]	AkhaCR
		Bread	Processed food	Moderate[a]	LisuCR
		Bread	Processed food	Strong[a]	LahuCR
		Meatball	Processed food	Moderate[a]	LahuCR
		Meatball	Processed food	Moderate[a]	LisuCR
		Sausage	Processed food	Strong[a]	LahuCR
		Sausage	Processed food	Strong[a]	LisuCR
	C.coccoides	Fresh Meat	Non-processed food	Moderate[a]	AkhaCR
	C.minuta	Noodle	Non-processed food	Moderate[b]	AkhaCR
	F.prausnitzii	Fresh Meat	Non-processed food	Strong[a]	AkhaCR
		Vegetable	Non-processed food	Moderate[a]	LisuCR
		Milk	Non-processed food	Moderate[b]	LisuCR
	A.muciniphila	Sticky rice	Non-processed food	Moderate[a]	LisuCR
		Vegetable	Non-processed food	Strong[b]	LahuCR
		Noodle	Non-processed food	Moderate[a]	LisuCR
		Herb	Non-processed food	Moderate[b]	LisuCR
		Bread	Processed food	Moderate[a]	LisuCR
		Sausage	Processed food	Moderate[a]	LisuCR
	Methanogens	Herb	Non-processed food	Moderate[a]	AkhaCR
	M.smithii	Fresh Meat	Non-processed food	Moderate[a]	AkhaCR
		Energy drink	Processed food	Strong[a]	AkhaCR

[a]: Positive correlation; [b]: Negative correlation
