# Supplementary material for: Effects of ethnicity and geography on the fecal microbiota and dietary habits of Tibeto-Burman hill tribes in Northern Thailand
Source: PLoS One. 2025 Oct 6;20(10):e0332108. doi: 10.1371/journal.pone.0332108 (PMC12500092; doi:10.1371/journal.pone.0332108)
Supplement: S2 Text — S1 Table. Primer pairs targeting bacterial 16S rRNA genes. S2 Table. Mean differences in gut microbiota abundance between ethnic groups within Chiang Mai Province. S3 Table. Mean differences in gut microbiota abundance between ethnic groups within Chiang Rai Province. S4 Table. Median differences in dietary consumption frequencies between ethnic groups within Chiang Mai Province. S5 Table. Median differences in dietary consumption frequencies between ethnic groups within Chiang Rai Province. S6 Table. Significant Spearman’s rank Correlations (p < 0.05) between gut microbiota and dietary habits in Chiang Mai province. S7 Table. Significant Spearman’s rank Correlations (p < 0.05) between gut microbiota and dietary habits in Chiang Rai province. S8 Table. Mean differences in gut microbiota abundance between geographic locations within the Akha ethnic group. S9 Table. Mean differences in gut microbiota abundance between geographic locations within the Lahu ethnic group. S10 Table. Median differences in dietary consumption frequencies between geographic locations within the Akha ethnic group. S11 Table. Median differences in dietary consumption frequencies between geographic locations within the Lahu ethnic group. (ZIP) [file pone.0332108.s002.zip › S9_Table.pdf]

S9 Table. Mean differences in gut microbiota abundance between geographic locations within the Lahu ethnic group.

**Bacterial**	**LahuCM (n=19)**	**LahuCR (n=16)**	***q*-value**
Firmicutes[a]	8.41±0.44	9.42±0.36	$q < 0.001$***
Bacteroidetes[a]	9.32±0.72	10.53±0.52	$q < 0.001$***
Actinobacteria[a]	7.74±0.60	8.69±0.55	$q < 0.001$***
Gammaproteobacteria[a]	7.25±0.31	7.33±0.37	0.534
Bacteroides[a]	10.15±0.72	11.31±0.56	$q < 0.001$***
Prevotella[a]	10.5±0.61	11.51±0.71	$q < 0.001$***
Streptococcus[a]	10.93±0.71	10.78±0.78	0.592
Enterococcus[a]	8.34±0.57	8.31±0.51	0.862
Roseburia[a]	8.43±0.66	9.22±0.60	0.001**
Ruminococcus[b]	9.16±0.75	9.7±0.42	0.048*
Coprococcus[a]	9.95±0.83	10.55±0.75	0.047*
Staphylococcus[a]	6.89±0.36	6.78±0.4	0.482
Osilobacterium[b]	6.64±1.00	7.13±2.06	0.073
Fusobacterium[b]	6.65±1.13	6.64±0.88	0.843
Actinomyces[a]	7.88±0.49	8.62±0.51	$q < 0.001$***
Bifidobacterium[a]	6.99±0.99	8.32±0.86	$q < 0.001$***
Lactobacillus[a]	7.38±0.62	8.41±0.63	$q < 0.001$***
B.fragilis[a]	6.96±0.88	8.61±1.15	$q < 0.001$***
C.coccoides[b]	8.53±0.57	9.18±0.46	0.002**
C.minuta[a]	8.85±0.61	9.52±0.52	0.002**
F.prausnitzii[b]	8.38±0.65	8.99±0.56	0.004**
A.muciniphila[b]	5.21±0.88	6.03±0.5	0.001**
Methanogens[b]	8.77±0.76	9.58±0.36	0.002**
M.smithii[b]	8.44±1.27	8.9±0.74	0.305

The relative abundance of gut microbiota is expressed as mean ± standard deviation (SD).

[a] Student's t-test with Benjamini–Hochberg method

[b] Mann-Whitney U with Benjamini–Hochberg method

*$q < 0.05$; **$q < 0.01$; ***$q < 0.001$
