# Supplementary material for: Effects of ethnicity and geography on the fecal microbiota and dietary habits of Tibeto-Burman hill tribes in Northern Thailand
Source: PLoS One. 2025 Oct 6;20(10):e0332108. doi: 10.1371/journal.pone.0332108 (PMC12500092; doi:10.1371/journal.pone.0332108)
Supplement: Attachment — Submitted filename: Response to Reviewers.pdf [file pone.0332108.s005.pdf]

Editor

1. Please ensure that your manuscript meets PLOS ONE's **style requirements**, including those for file naming.

Response: We have carefully reviewed the manuscript and supporting files to ensure that all formatting and file naming conventions comply with PLOS ONE's style requirements, as outlined in the journal's guidelines.

2. Please note that **funding information** should not appear in any section or other areas of your manuscript. We will only publish funding information present in the Funding Statement section of the online submission form. Please remove any funding-related text from the manuscript.

Response: We have removed all funding-related information from the manuscript and have provided the relevant details exclusively in the Funding Statement section of the online submission form, in accordance with the journal's requirements.

3. PLOS requires an **ORCID iD** for the **corresponding author** in Editorial Manager on papers submitted after December 6th, 2016. Please ensure that you have an ORCID iD and that it is validated in Editorial Manager. To do this, go to 'Update my Information' (in the upper left-hand corner of the main menu), and click on the Fetch/Validate link next to the ORCID field. This will take you to the ORCID site and allow you to create a new iD or authenticate a pre-existing iD in Editorial Manager.

Response: We appreciate the reminder. We have successfully verified the corresponding author's ORCID iD and updated our information in Editorial Manager as instructed.

4. We note that **Figure 1** in your submission contain map/satellite images which may be **copyrighted**. All PLOS content is published under the Creative Commons Attribution License (CC BY 4.0), which means that the manuscript, images, and Supporting Information files will be freely available online, and any third party is permitted to access, download, copy, distribute, and use these materials in any way, even commercially, with proper attribution. For these reasons, we cannot publish previously copyrighted maps or satellite images created using proprietary data, such as Google software (Google Maps, Street View, and Earth).

Response: We were unable to obtain permission from the original copyright holder to publish the map/satellite images in Figure 1 under the CC BY 4.0 license. Therefore, we have removed Figure 1 and replaced it with Table 1, which presents the relevant sampling information.

5. Please review your **reference list** to ensure that it is complete and correct. If you have cited papers that have been retracted, please include the rationale for doing so in the manuscript text, or remove these references and replace them with relevant current references. Any changes to the reference list should be mentioned in the rebuttal letter that accompanies your revised manuscript. If you need to cite a retracted article, indicate the article's retracted status in the References list and also include a citation and full reference for the retraction notice.

Response: We have carefully reviewed our reference list. All references remain current, relevant, and valid for the scientific context of our study.

Reviewer 1

This manuscript presents a comprehensive and well-performed study investigating influences of ethnicity and geography on gut microbiota composition and dietary intake with respect to Tibeto-Burman hill-tribe populations in Northern Thailand. Quantitative PCR, multivariate statistics (MFA, PLS-DA), and the sampling from carefully defined ethnic and geographic subgroups lend weight to this field. The manuscript is straightforward, the figures informative, and the statistical approaches proper. However, there are issues that should be addressed to strengthen the manuscript prior to publication.

Response: We are grateful for the reviewer's thoughtful feedback and careful evaluation of our manuscript. We sincerely thank you for the insightful and constructive comments and suggestions, which have significantly helped us improve the overall structure and clarity of the manuscript. Below, we provide a point-by-point response to each comment.

Comments

1. While qPCR could be considered an exact technique-matters for numbers, the authors would be wise to elucidate why they opted for qPCR instead of the more common 16S rRNA sequencing, especially concerning taxonomic range and resolution. Were there any drawbacks with taxonomic profiling?

Response: We thank the reviewer for this insightful comment. As this study aimed to quantify and compare specific gut bacteria of interest across different ethnic groups and geographic locations, we employed a qPCR-based approach, which enabled rapid detection with high sensitivity and specificity (Yan et al., 2024). This method provided a preliminary overview of how ethnic and geographic factors may influence gut microbiota in these underrepresented communities. While qPCR provides high sensitivity and specificity for selected taxa, we

acknowledge that qPCR does not provide a comprehensive overview of the microbial community structure or diversity, as would be achievable through 16S rRNA gene sequencing, which offers broader taxonomic resolution. We have discussed this as a limitation of our study in the Discussion section (lines 661-666).

2. A total of 102 subjects divided into various subgroups (3 ethnic groups × 2 locations) renders per-group samples quite small for statistically conclusive interpretations. Please consider conducting a power analysis or discussing the limitations presented by such small group sizes.
Response: We appreciate the reviewer's comment regarding the small sample size within each subgroup and its implications for statistical interpretation. We conducted a power analysis for group comparisons using the *pwr* package in R. The analysis indicated that approximately 64 participants per group (for two-group comparisons) and 52 participants per group (for three-group comparisons) would be required to detect a medium effect size with 80% power at a significance level of 0.05. However, recruitment for this study was based on voluntary participation from specific communities, namely the Tibeto-Burman hill tribes, which resulted in a smaller sample size than the calculated requirement. This limitation, which stems from reliance on voluntary participation within a specific population, has been acknowledged in the revised manuscript (lines 651-654).

3. The manuscript tends to assert causal relationships between geography and gut microbiota composition. However, strong causal claims are not supportable due to the cross-sectional design of the study. It is requested that the language state association rather than causality.
Response: We greatly appreciate the reviewer's thoughtful comment. We agree that, given the cross-sectional design of our study, the language should reflect associations rather than causal relationships. In response to this valuable suggestion, we have carefully revised the language throughout the manuscript to ensure that statements regarding geography and gut microbiota composition are presented as associations rather than implying causality.

4. Environmental exposures beyond what has been considered in diet and elevation have not been discussed (i.e., water sources, sanitation, occupational activities). These exposures could alter the gut microbiota and potentially confound effects of geography. Consider mentioning this as a limitation.
Response: Thank you for raising this important point. We agree that environmental exposures beyond diet and elevation, such as water sources, sanitation, and occupational activities, may

serve as potential confounders influencing gut microbiota composition. We have acknowledged this as a limitation of our study and highlighted the importance of incorporating these variables in future research (lines 658-661).

5. The authors state that all relevant data are available in the manuscript and supporting information. The authors should also please consider making raw qPCR data and all metadata used in the multivariate analyses

Response: Thank you for your valuable suggestion. We have provided raw qPCR data and all relevant metadata used in the multivariate analyses in S1-S4 Files.

6. Clarify how the participants were confirmed to have no kinship relation and to be representative of their ethnic groups.

Response: Thank you for raising this important point. We confirmed that participants had no close kinship relationships and were representative of their respective ethnic groups through a sampling process aligned with a previous study (Kampuansai et al., 2023). The selection of ethnic groups was guided by historical and ethnographic data, including detailed accounts of cultural identity and community organization from Inta et al., 2023, as well as databases from the Health Center for Ethnic Group, Marginal People and Migrant Worker (HHDC), and the Princess Maha Chakri Sirindhorn Anthropology Centre (SAC). Participant recruitment involved direct interviews with volunteers and consultations with local community leaders who were knowledgeable about family lineages and community structure. This process ensured that participants were not closely related to others in the study for at least three generations. We have clarified this procedure in the revised *Materials and Methods* section (lines 112-116).

7. The manuscript also states that illiterate participants signed a consent form with a fingerprint. Please specify if this procedure is aligned with national and international procedures for ethical consideration.

Response: Thank you for highlighting this important point. We confirm that the procedure for obtaining informed consent from illiterate participants using fingerprint signatures strictly adhered to all relevant ethical guidelines and regulations, in accordance with the Declaration of Helsinki. The consent form was verbally explained to each participant in their native language, and an impartial witness was present throughout the process. The witness also signed a statement confirming that the participant understood the information and voluntarily agreed

to participate. We have expanded the *Materials and Methods* section to clarify this procedure (lines 100-105) and have uploaded the translated consent form for reference.

8. Heatmaps and similar figures (e.g., Figs 2 and 3) require better contrast and simpler and clearer legends to be more interpretable.

Response: We have updated the figure numbers, which are now presented as Figure 1–2. In addition, we have simplified and clarified the figure legends to improve their interpretability for readers.

9. Use the same ethnic group names consistently throughout (e.g., "AkhaCM" vs. "Akha CM") and explain the abbreviations at first use both in the text and in figure legends.

Response: The ethnic group names in the revised manuscript have been corrected for consistency throughout the text, and all abbreviations have been clearly explained at their first occurrence in both the main text and figure legends.

10. Some sentences could be clearer (e.g., lines 295–298). A thorough proofreading is recommended.

Response: We thank the reviewer for the suggestion. The sentences have been revised to improve clarity and readability, as recommended (lines 316-319). Additionally, the manuscript has undergone professional proofreading by Editage. An editing certificate has been included for your reference.

Reviewer 2

I have carefully reviewed the manuscript titled "Effects of ethnicity and geography on the fecal microbiota and dietary habits of Tibeto-Burman hill tribes in Northern Thailand" submitted to Plos One. The authors have presented a well-structured and scientifically sound study that contributes valuable insights to the field of Gut Microbiome Study.

Strengths of the Manuscript:

• The research question is clearly defined and relevant.
• The methodology is appropriate and well executed.
• Data analysis is thorough and supports the conclusions drawn.
• The discussion effectively contextualizes the findings with existing literature.
• The manuscript is well written and easy to follow.

Recommendation: I recommend acceptance of this manuscript in its current form / with minor editorial revisions (grammar, formatting), if needed by the journal.
Congratulations to the authors on their work.

Response: We greatly appreciate the reviewer for your thorough evaluation and kind feedback. We are grateful for the positive comments regarding the clarity of our research question, the appropriateness of our methodology, and the strength of our analysis and discussion. To ensure clarity and consistency, the manuscript has been professionally proofread by Editage A certificate of editing has been included as an attachment for your reference. Additionally, the formatting has been revised to ensure consistency, as previously mentioned by the editor.

Informed Consent Document for Genetic Research

I, ___________________________________, have decided to participate in the research project "Genetic adaptation to high-altitude and evergreen forest environmental selection in the hill-tribe group of Thailand," which will involve the use of my blood and stool samples for genetic research.

I have received comprehensive information and explanations regarding the aforementioned genetic research and have had the opportunity to ask questions, all of which were answered to my satisfaction. I have also had enough time to read and understand the study's details and make an informed decision about using my blood and stool samples.

I acknowledge that I can freely refuse to participate in this genetic research without affecting my medical care, my existing rights, or my participation in any clinical research. Furthermore, I can change my decision at a later time.

Participant's Signature: __________________ Date: _______

(__________________)

................(For participants who are unable to read but can understand spoken language)................

I am unable to read, but the researcher has read the information sheet and this consent form to me, and I fully understand its contents. I am willingly signing or placing my fingerprint on this consent form to participate in this genetic research.

Participant's fingerprint: ______________________ Date: _______

(___________________)

Researcher's Signature: __________________ Date: _______

(__________________)

Statement of an impartial witness

I was present during the informed consent process and confirmed that the person obtaining consent has read and/or explained the information sheet to __________________________. The individual named above was given the opportunity to ask questions and voluntarily agreed to participate in the research after fully understanding the information as presented in this document.

Witness's Signature: __________________ Date: _______

(__________________)
